# Clinical Outcomes of Non-Elastic Compression Bandage Versus Elastic Bandage Following Lateral Ankle Ligament Repair

**DOI:** 10.3390/healthcare13101182

**Published:** 2025-05-19

**Authors:** Jie Yang, Guocheng Ding, Zhixin Duan, Yixiang Yan, Yuyue Zhang, Dong Jiang, Jianquan Wang

**Affiliations:** 1 Department of Sports Medicine, Peking University Third Hospital, Institute of Sports Medicine, Peking University, Beijing 100191, China; yangjie0704@icloud.com (J.Y.); 13603393009@163.com (G.D.); 2110301311@stu.pku.edu.cn (Z.D.); 2210301151@stu.pku.edu.cn (Y.Y.); 2410301339@stu.pku.edu.cn (Y.Z.); 2 Beijing Key Laboratory of Sports Injuries, Beijing 100191, China; 3 Engineering Research Center of Sports Trauma Treatment Technology and Devices, Ministry of Education, Beijing 100191, China

**Keywords:** lateral ankle ligament repair, elastic bandage, cotton padding, non-elastic compression bandage, postoperative rehabilitation

## Abstract

**Objectives**: This study aims to compare the postoperative clinical outcomes of using non-elastic compression bandages versus elastic bandages after lateral ankle ligament repair. **Methods**: This retrospective study analyzed a total of 110 patients who underwent repair surgery for chronic lateral ankle ligament injuries. Based on the postoperative bandaging method, patients were divided into two groups: the non-elastic compression bandage group (Group NECB, 55 cases) and the elastic bandage group (Group EB, 55 cases). A comparison was made between the two groups of patients regarding postoperative ankle joint swelling, pain scores (VAS scores), ankle function (AOFAS Ankle–Hindfoot Scale), range of motion of the ankle joint, the incidence of perioperative complications (including subcutaneous ecchymosis, wound seepage, and events requiring loosening of the bandage due to pain), and the status of return to work postoperatively. **Results**: There were no significant differences between the two groups in terms of early postoperative ankle joint swelling or increased circumference (0.53 ± 1.47 cm vs. 1.08 ± 1.84 cm, *p* = 0.095) or VAS scores at 1 day (3.84 ± 2.14 vs. 3.63 ± 2.03, *p* = 0.595), 7 days (2.20 ± 1.89 vs. 1.78 ± 1.67, *p* = 0.216), 14 days (1.45 ± 1.56 vs. 0.97 ± 1.23, *p* = 0.075), or 3 months (1.27 ± 1.50 vs. 1.38 ± 1.76, *p* = 0.744). Both groups demonstrated comparable functional recovery based on AOFAS scores at 3 months (89.89 ± 8.08 vs. 90.05 ± 9.50, *p* = 0.926), ROM in all measured directions (*p* > 0.05), and return to work status (*p* = 0.567). However, the incidence of complications was significantly lower in Group NECB (3.6%) compared to Group EB (30.9%). The reported complications in Group EB were mainly related to postoperative subcutaneous ecchymosis and discomfort requiring bandage loosening. **Conclusions**: There is no difference between non-elastic compression bandaging with cotton padding and elastic bandaging in postoperative swelling, pain, and functional recovery. However, in reducing the incidence of postoperative skin adverse events, using non-elastic compression bandages with cotton padding proves to be more ideal as a bandaging method after lateral ankle ligament repair.

## 1. Introduction

Lateral ankle ligament injuries are common sports-related injuries, particularly during activities involving running, jumping, and various ball games [1]. Such injuries not only cause pain and restricted movement but may also lead to a series of long-term issues [2,3]. If improperly managed, this type of injury can result in ankle instability and subsequent joint degeneration, which directly impacts athletic performance and may lead to chronic ankle instability [4]. Furthermore, unilateral chronic ankle instability may lead to proprioceptive deficits, which in turn cause a reorganization of the sensorimotor system, ultimately resulting in altered postural control during single-leg stance on the healthy limb [4,5]. Effective treatment of lateral ankle ligament injuries and postoperative rehabilitation management are thus critically important. Ankle lateral ligament repair surgery has become an effective treatment for restoring ankle stability, reducing pain, and preventing long-term complications [6,7]. Meanwhile, postoperative management during the recovery period following ligament repair surgery, particularly the selection of appropriate bandaging methods, plays a critical role in accelerating the speed and quality of patient recovery [8]. The use of elastic bandages after surgery can provide beneficial effects in reducing swelling [9]. Research has indicated that improper application of elastic bandages can lead to varying degrees of postoperative complications, such as subcutaneous ecchymosis resulting in skin discoloration, limb swelling, numbness, and joint effusion [10]. Enhanced observation and scientifically effective postoperative management of bandaging in patients undergoing ankle joint surgery are essential measures for preventing complications and facilitating timely rehabilitation.

In traditional postoperative management of ankle ligament injuries, elastic bandaging has been widely adopted due to its simplicity and low cost [9]. However, patients often report more discomfort during the use of elastic bandages [10]. Our study proposes the use of non-elastic compression bandages with cotton padding as an alternative to elastic bandages, as this non-elastic bandage may provide a more comfortable experience for patients. The current literature lacks direct comparison of non-elastic versus elastic bandaging in the context of orthopedic postoperative care, particularly for lateral ankle ligament repair. This study, therefore, designed a retrospective analysis primarily aimed at comparing the differences in clinical efficacy of non-elastic cotton-padded compression bandages versus elastic bandages following lateral ankle ligament repair in promoting postoperative recovery. Specifically, we focused on the impact of these two bandaging methods on postoperative ankle edema, pain control, recovery of ankle function, range of motion, and the incidence of complications such as subcutaneous bruising, wound bleeding, and adjustments due to discomfort. We hypothesize that patients using the non-elastic cotton-padded compression bandage may exhibit better clinical outcomes.

## 2. Materials and Methods

### 2.1. Study Design and Participants

This retrospective analysis included patients who underwent lateral ankle ligament repair at our hospital between December 2023 and June 2024. The study protocol was reviewed and approved by the hospital ethics committee (protocol code: S2018133; date of approval: 9 March 2018), and all patients signed informed consent forms. The study strictly protected patient confidentiality, ensuring objectivity and scientific validity. A total of 110 eligible patients were included in the study. Inclusion criteria were as follows: clinical and imaging-confirmed chronic lateral ankle ligament injury, age between 18 and 60 years, first-time ankle ligament repair surgery, and a minimally invasive incision length of 3–4 cm. Exclusion criteria included significant injuries to other structures in the ankle, severe cardiovascular disease, blood disorders, or other major systemic diseases, as well as failure to follow the prescribed postoperative bandaging or monitoring requirements. Patients were assigned to one of two groups based on the postoperative bandaging method: Group NECB (non-elastic compression bandage with cotton padding, 55 cases) and Group EB (elastic bandage, 55 cases). All patients in this study had chronic lateral ankle ligament injuries, with a history of repeated sprains or discomfort after intense activities, and had injuries for over three months. In all surgeries, a minimally invasive technique with a 3–4 cm arcuate incision was employed to repair the damaged ligaments.

### 2.2. Postoperative Bandaging Methods

**Figure 1:**
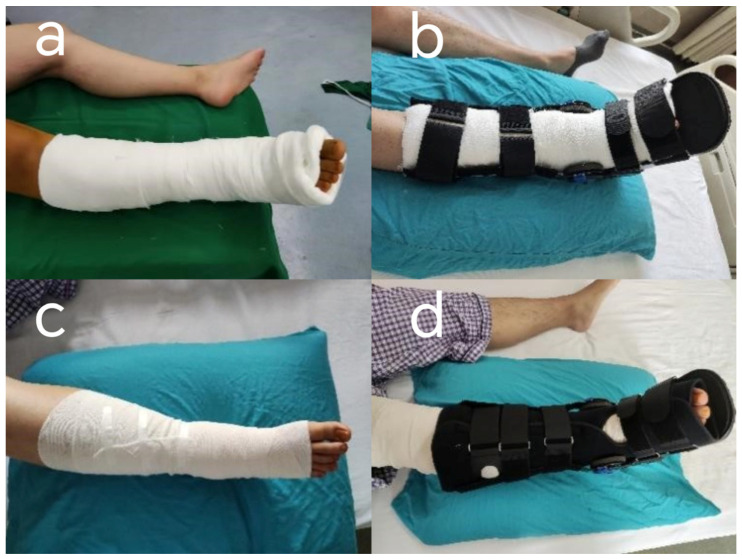
(**a**) Cotton padding non-elastic bandaging. (**b**) Cotton padding non-elastic bandaging with brace fixation. (**c**) Elastic bandaging. (**d**) Elastic bandaging with brace fixation.

All surgeries were performed by two experienced senior surgeons using the same surgical technique. And the patient enrollment and bandaging were also performed by these two surgeons and their assistants. Group NECB used non-elastic compression bandages with cotton padding, in which cotton padding was applied to the ankle skin postoperatively, followed by a non-elastic bandage to provide compression, and an external ankle brace was applied for fixation [11]. Group EB used elastic bandages, applying an elastic bandage in a figure-eight and spiral pattern over the surgical area, with the tension adjusted according to the patient’s ankle circumference (Figure 1) [12]. All dressings were removed during suture removal on postoperative day 14.

### 2.3. Outcome Measures

**Figure 2:**
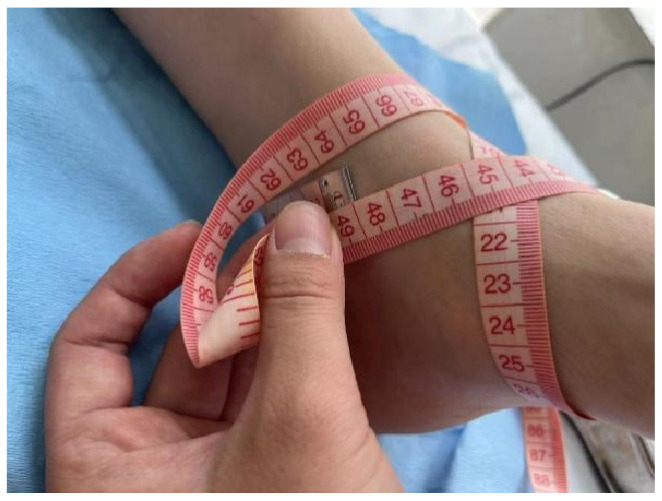
Method of edema assessment: With the ankle in a neutral position, the zero point of the tape measure is placed in the groove at the edge of the lateral malleolus, between the lateral malleolus and the tibialis anterior tendon, looping medially over the sole to the base of the fifth metatarsal, then around the inner ankle in a figure-eight, passing under the Achilles tendon and below the lateral malleolus, and finally meeting the starting point.

Primary outcomes included ankle edema at two weeks postoperatively (measured by maximum ankle circumference) (Figure 2); pain scores at 1 day, 7 days, 14 days, and 3 months postoperatively (using the Visual Analog Scale, VAS); the American Orthopaedic Foot & Ankle Society (AOFAS) Ankle–Hindfoot Scale score at 3 months postoperatively; the range of motion of the ankle joint (angles of dorsiflexion, plantarflexion, inversion, and eversion) at 3 months postoperatively; the incidence of postoperative complications (including subcutaneous bruising, wound bleeding, and events requiring adjustment of bandage tightness due to discomfort); and whether the patient returned to work at 3 months postoperatively [13,14]. All evaluation indicators were assessed at 1 week, 2 weeks, and 3 months postoperatively.

### 2.4. Data Collection and Statistical Analysis

Data were analyzed using IBM SPSS Statistics 22.0 software. Continuous variables were expressed as mean ± standard deviation ($\bar{x}$ ± sd), and comparisons between groups were performed using the independent samples *t*-test, with t-values, effect sizes (Cohen’s d), and 95% confidence intervals (CIs) reported. Categorical data were expressed as percentages, with group comparisons conducted using the χ^2^ test. A *p*-value of <0.05 was considered statistically significant. Effect sizes were interpreted as small (d ≈ 0.2), medium (d ≈ 0.5), or large (d ≥ 0.8) based on conventional thresholds. All statistical tests were two-tailed, and homogeneity of variance was confirmed via Levene’s test prior to *t*-test analysis. To ensure statistical power, the sample size was estimated based on two primary outcome measures: the VAS for pain and the AOFAS Scale. With a significance level (α) of 0.05, power (1-β) of 80%, and two-tailed independent samples *t*-test, the calculated required sample size was 44 per group. For the AOFAS score, an intergroup difference of 5 points (SD = 9) under identical conditions yielded a required sample size of 51 per group. Accounting for potential data loss or dropout, we ultimately enrolled 55 participants per group (total n = 110) to ensure adequate statistical power and robust results.

## 3. Results

### 3.1. Patient Demographics

A total of 110 patients with lateral ankle ligament injuries were included, divided into Group NECB (non-elastic compression bandage with cotton padding) and Group EB (elastic bandage), with 55 cases in each group. There were no significant differences in age, gender, injury side, or BMI between the groups (*p* > 0.05). See Table 1 for specific data.

### 3.2. Postoperative Ankle Pain Scores (VAS Scores)

There was no significant difference in pain scores between the two groups at 1 day postoperatively. At 7 days, 14 days, and 3 months postoperatively, the pain scores in both groups still showed no significant difference (*p* > 0.05). The specific data can be found in Table 2.

### 3.3. Postoperative Joint Swelling and Complications

**Figure 3:**
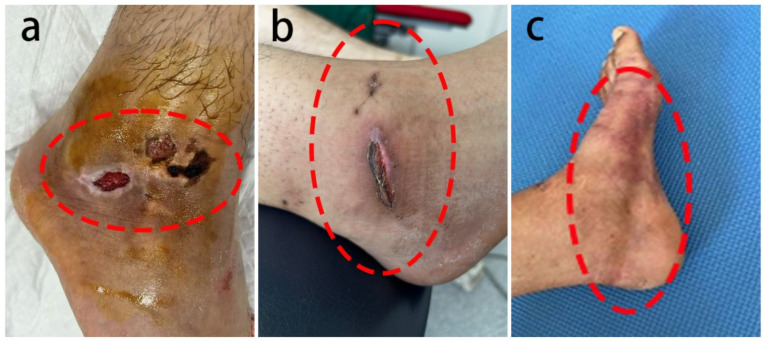
Adverse events: (**a**,**b**) skin healing issues two weeks postoperatively in the elastic bandage group and (**c**) significant subcutaneous bruising in the elastic bandage group.

At 2 weeks postoperatively, the ankle joint swelling showed no significant difference in the degree of edema between the two groups (*p* > 0.05). A comparison of the incidence of complications (including subcutaneous ecchymosis, wound seepage, and pain requiring adjustment of bandage tightness) within 2 weeks postoperatively revealed that the adverse event rate in Group NECB was significantly lower than that in Group EB (Figure 3). The specific data can be found in Table 3.

### 3.4. Postoperative AOFAS Ankle–Hindfoot Scale Scores

Preoperatively, both groups were assessed using the AOFAS Ankle–Hindfoot Scale, with no significant difference in scores between the two groups. At 3 months postoperatively, both groups showed significant improvements in their AOFAS scores compared to preoperative levels, and there was still no significant difference between the two groups. Detailed data can be found in Table 4.

### 3.5. Postoperative Ankle Joint Range of Motion

At 3 months postoperatively, we measured the range of motion (ROM) of the ankle joint in dorsiflexion, plantarflexion, inversion, and eversion for both groups, and calculated the difference between the actual and normal range of motion. The analysis revealed no significant differences between the two groups in the range of motion in any of the four directions at 3 months postoperatively (*p* > 0.05). Detailed data are presented in Table 5.

### 3.6. Postoperative Work Return Status

At 3 months postoperatively, 49 patients in Group NECB had returned to work, while 6 had not. In Group EB, 47 patients had returned to work, and 8 had not. There was no significant difference between the two groups regarding return to work status (*p* = 0.567).

## 4. Discussion

This study found that there is no significant difference between Group NECB, which used non-elastic compression bandaging, and Group EB, which used elastic bandaging, in terms of postoperative ankle swelling control, pain reduction, recovery of ankle function, range of motion, or return to work. However, Group NECB demonstrated a significantly lower incidence of postoperative skin-related adverse events compared to Group EB. This suggests that the application of a cotton pad non-elastic pressure bandage in ankle lateral ligament repair surgery offers certain clinical advantages.

Postoperative control of adverse events in the ankle is crucial for patients’ early and rapid recovery [15,16]. Group NECB exhibited a significantly lower incidence of complications compared to Group EB. We speculate that this may be attributed to the stable pressure and gradual reduction in compression provided by the non-elastic cotton bandage, which could promote wound healing, lower infection risk, and reduce adverse skin reactions. In Group EB, patients reported more delayed, extensive subcutaneous bruising, indicating that clinicians should ideally apply padding uniformly or moderate the pressure when using elastic compression bandages to help distribute pressure. Notably, two cases of poor wound healing were reported in Group EB, highlighting the potential for elastic bandages to induce more severe adverse skin reactions, such as skin breakdown. These results indicated that Group NECB patients experienced superior skin protection in the ankle surgery area compared to Group EB.

Regarding ankle joint edema, no significant difference in ankle edema was observed between Group NECB and Group EB. The cotton-padded non-elastic bandage may initially prevent swelling, but as swelling resolves in the first postoperative week, reduced pressure between the skin and padding likely enhances blood circulation and lymphatic drainage, minimizing local adverse skin reactions. Studies indicate that the moldable nature of cotton padding better conforms to the anatomical structure of the ankle, allowing it to accommodate postoperative changes in swelling and providing effective support and protection [17,18]. In contrast, the elastic bandage exerts continuous external pressure on the surgical area, potentially affecting microcirculation in the later stages of recovery. The tension of an elastic bandage depends on its elongation during application. When used postoperatively, it can effectively compress limbs, reducing joint effusion, providing hemostasis, alleviating swelling and pain, and preventing venous thrombosis if applied with appropriate pressure [19,20,21]. However, the clinician must ensure that the tension is suitable: too loose may lead to joint cavity bleeding or effusion, while too tight can impede peripheral blood circulation [22,23]. Therefore, postoperatively, close monitoring of peripheral circulation is essential to detect swelling or numbness. Patients should promptly report pain to enable timely etiology analysis and symptomatic treatment. Additionally, measures should be taken to prevent iatrogenic events, such as compartment syndrome or ischemic muscle contracture, caused by excessively tight bandaging.

In terms of pain control, VAS scores in both groups decreased significantly postoperatively, with no notable differences between them at any time point. The method of bandaging can directly influence the patient’s comfort level and perception of pain. Ensuring patient comfort during the postoperative period can facilitate enhanced local blood circulation, thereby promoting the dissipation of inflammatory mediators and accelerating tissue repair [24]. In terms of functional recovery, both groups showed significant improvements in AOFAS scores and near-normal ankle range of motion at three months postoperatively, with no significant differences between them. Most patients in both groups returned to work. However, Group NECB reported greater joint comfort, suggesting non-elastic bandaging may enhance postoperative recovery. All these indicators align with the goal of early postoperative rehabilitation, as early functional recovery is a critical objective in the postoperative management of lateral ankle ligament repair [13,25].

The findings of our study demonstrated that non-elastic compression bandages significantly reduced postoperative complications compared to elastic bandages without compromising functional outcomes. A rehabilitation study on calcaneal fractures post-surgery, which used a similar bandaging method, found that this bandaging approach is an effective method for postoperative soft tissue management [11]. However, some studies contrast with our findings. For example, a review pointed out that the use of non-elastic bandages or medical elastic compression stockings does not provide benefits for postoperative edema treatment in orthopedic surgery [9]. These discrepancies may stem from variations in specific bandaging methods and different surgeries. Although this study compared the clinical differences between elastic and non-elastic bandages following lateral ankle ligament repair, Matthews et al.’s study found that, after primary total knee arthroplasty, the use of elastic bandages did not provide additional clinical benefits compared to not using a bandage [26]. Considering that bandages may offer similar effects in orthopedic surgeries, this suggests that future research may need to include a no-bandage group to better understand the clinical benefits of elastic and non-elastic bandages after lateral ankle ligament repair. However, they also found that, after post-TKA surgery, the total wound complication rate was 6% in the elastic bandage group versus 12% in the no-bandage group [26]. Moreover, some studies have indicated that compression therapy after ankle fracture surgery is beneficial for swelling, pain, and ankle joint range of motion [27].

This study has several limitations. Firstly, the follow-up period was limited to three months, which may not capture long-term complications or differences in functional outcomes. Secondly, as this study was conducted at a single center with standardized surgical procedures, the generalizability of the findings to other populations or healthcare settings may be limited. Future studies involving longer follow-up and more diverse clinical environments are needed to validate and expand upon these results.

In summary, this study demonstrates that non-elastic compression bandages with cotton padding have clinical advantages over traditional elastic bandages in preventing adverse skin events after lateral ankle ligament repair. This includes fewer skin complications. In clinical practice, cotton-padded non-elastic bandages may be considered a preferred method of bandaging for postoperative management of lateral ankle ligament repairs. Future research should further explore optimal pressure levels, pressure duration, and combined effects with other postoperative management measures, such as cryotherapy and limb elevation, to optimize postoperative recovery protocols, enhance patient satisfaction, and improve quality of life.

## 5. Conclusions

There is no difference between non-elastic compression bandaging with cotton padding and elastic bandaging in postoperative swelling, pain, and functional recovery. However, in the incidence of postoperative skin adverse events, using non-elastic compression bandages with cotton padding proves to be more ideal as a bandaging method after lateral ankle ligament repair.

## Figures and Tables

**Table 1:** Comparison of baseline information between the two patient groups.

	Group NECB (n = 55)	Group EB (n = 55)	*t*	*p*	Effect Sizes	95% CI
Age	33.18 ± 10.49	30.44 ± 9.87	1.41	0.163	0.27	(−1.15, 6.63)
Sex (Male/Female)	30/25	28/27		0.704		
BMI	24.11 ± 3.39	25.15 ± 4.20	−1.40	0.164	0.27	(−2.50, 0.42)
Injured side (Left/Right)	26/29	32/23		0.252		

**Table 2:** Comparison of postoperative pain scores (VAS scores) between the two patient groups.

Time Point	Group NECB VAS Score	Group EB VAS Score	*t*	*p*	Effect Sizes	95% CI
1 day	3.84 ± 2.14	3.63 ± 2.03	0.53	0.595	0.10	(−0.56, 0.98)
7 days	2.20 ± 1.89	1.78 ± 1.67	1.25	0.216	0.24	(−0.26, 1.10)
14 days	1.45 ± 1.56	0.97 ± 1.23	1.80	0.075	0.34	(−0.05, 1.01)
3 months	1.27 ± 1.50	1.38 ± 1.76	−0.33	0.744	0.07	(−0.69, 0.49)

**Table 3:** Postoperative edema at 2 weeks, delay in the initiation of rehabilitation, and adverse events.

	Difference in Ankle Circumference	Number of Adverse Events (e.g., Hematoma, Dressing Change)
Group NECB	0.53 ± 1.47	Poor wound healing: 0 subcutaneous ecchymosis: 1 Dressing adjustment required: 2
Group EB	1.08 ± 1.84	Poor wound healing: 2 subcutaneous ecchymosis: 6 Dressing adjustment required: 9
*t*	−1.69	
*p*	0.095	
Effect Sizes	0.32	
95% CI	(−1.21, 0.11)	

**Table 4:** Comparison of AOFAS Ankle–Hindfoot Scale scores between the two groups.

	Group NECB	Group EB	*t*	*p*	Effect Sizes	95% CI
preoperative	74.89 ± 9.12	75.12 ± 9.34	−0.13	0.782	0.02	(−3.62, 2.72)
3 months	89.89 ± 8.08	90.05 ± 9.50	−0.09	0.926	0.02	(−3.41, 3.09)

**Table 5:** Difference between postoperative ankle joint range of motion and normal range of motion at 3 months.

	Group NECB	Group EB	*t*	*p*	Effect Sizes	95% CI
dorsiflexion	2.16° ± 3.08°	2.43° ± 3.27°	−0.42	0.676	0.08	(−1.60, 1.06)
plantarflexion	0.86° ± 1.80°	1.42° ± 2.92°	−1.11	0.272	0.23	(−1.54, 0.42)
inversion	0.93° ± 1.90°	1.11° ± 2.66°	−0.37	0.711	0.08	(−1.16, 0.80)
eversion	0	0.11° ± 0.69°	−1.17	0.243	0.22	(−0.30, 0.08)

## Data Availability

The original contributions presented in this study are included in the article. Further inquiries can be directed to the corresponding authors.
